# Vacuum‐Nitrogen Assisted (VANS) Topotactical Deintercalation for Extremely Fast Production of Functionalized Silicene Nanosheets

**DOI:** 10.1002/smll.202406088

**Published:** 2024-12-18

**Authors:** Erika Kozma, Christian Martella, Anita Eckstein Andicsová, Sepideh Gharedaghi, Alessio Lamperti, Chiara Massetti, Andrej Opálek, Carlo Grazianetti, Francesco Galeotti, Alessandro Molle

**Affiliations:** ^1^ Istituto di Scienze e Tecnologie Chimiche “Giulio Natta” Consiglio Nazionale delle Ricerche Via A. Corti 12 Milano 20133 Italy; ^2^ Istituto per la Microelettronica e Microsistemi Consiglio Nazionale delle Ricerche Sede di Agrate Brianza Via C. Olivetti 2 Agrate Brianza I‐20864 Italy; ^3^ Polymer Institute of Slovak Academy of Sciences Dúbravská cesta 9 Bratislava 84541 Slovakia; ^4^ Institute of Materials and Machine Mechanics Slovak Academy of Sciences Dúbravská cesta 9 Bratislava 84513 Slovakia

**Keywords:** 2D materials, nanosheets, silicene flakes, topotactical deintercalation, VANS

## Abstract

Silicene, the analog of graphene composed of silicon atoms arranged in a honeycomb lattice, has garnered significant attention due to its unique properties, positioning it as a promising candidate for various applications in electronic devices, photovoltaics, photocatalysis, and biomedicals. While the chemical synthesis of silicene nanosheets has traditionally involved time‐spending and expensive‐ methods, this study introduces a rapid vacuum/nitrogen cycle assisted (VANS) protocol that dramatically speeds up the production of silicene. The strategic implementation of vacuum/nitrogen cycles provides the efficient removal of the generated hydrogen, boosting the overall reaction kinetics while maintaining inert reaction conditions to prevent oxidation. In contrast to previous methodologies, usually qualified by prolonged reaction durations of up to 5 days and low reaction temperatures (−30 °C), this integrated approach substantially shortens the synthesis time from hours to minutes. Indeed, the VANS method drastically reduces the reaction time, operates at room temperature, and exhibits the successful fabrication of silicene flakes with structural properties comparable to those achieved through prolonged reaction times and low temperatures.

## Graphical abstract

Silicene, an analog of graphene with silicon atoms arranged in a honeycomb lattice, shows promise for electronic devices and photocatalysis. A new rapid VANS (vacuum‐nitrogen assisted) protocol accelerates its production by efficiently removing hydrogen and maintaining inert conditions. Unlike traditional methods, VANS reduces synthesis time from days to minutes, producing silicene flakes with comparable properties at room temperature.

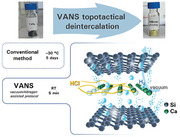

## 1 Introduction

In recent years, there has been a remarkable surge in the exploration of graphene, a material that has captivated scientists owing to its extraordinary properties.^[^ ^1^ ^]^ However, the landscape of 2D materials extends beyond graphene, and elemental analogs have emerged as significant contenders in the pursuit of advancements in nanotechnology.^[^ ^2^, ^3^, ^4^, ^5^, ^6^, ^7^ ^]^

Silicene, a fascinating 2D material comparable to graphene but composed of silicon atoms, has attracted significant attention for its potential applications in electronics and optoelectronics.^[^ ^8^ ^]^ However, the broader exploration and utilization of silicene is hindered by the expensive production techniques associated primarily with bottom‐up approaches like molecular beam epitaxy (MBE).^[^ ^9^ ^]^ In response, researchers are exploring more cost‐effective methods for synthesizing silicene, with topotactical deintercalation emerging as a promising approach. Bypassing the need for costly equipment and intricate processes associated with physical methods, this approach opens up new possibilities for large‐scale silicene synthesis eventually aiming at the manufacture of printable silicene inks in line with other 2D materials. Furthermore, this method provides greater flexibility in tailoring the properties of the resulting materials to meet specific application requirements in electronics, sensing, catalysis, molecular electronics, theranostics, and biomedicals.

Silicene synthesis via topotactical deintercalation is achieved through the direct transformation of layered CaSi_2_ Zintl phases, characterized by Si‐ layers separated by Ca^2+^ cations, which is chemically reactive with acids or electrophiles to yield covalently terminated Si─H, Si─OH, or SiR species.^[^ ^10^ ^]^ In the pioneering work of exfoliation, 2D silicon nanosheets, later characterized as siloxene (Si_6_H_3_(OH)_3_), were obtained in a two‐step reaction procedure.^[^ ^11^ ^]^ First, hexagonal “graphite‐like” platelets of CaSi_2_ were synthesized through the reaction of Na, CaCl_2_, and Si powder. Following this synthesis, the subsequent treatment with a variety of acids led to the formation of a distinctive yellow powder, characterized by an approximate stoichiometry of Si_8_O_4_H_6_. Later, a structural model for siloxene (Kautsky siloxane), characterized by isolated Si_6_ rings interconnected by Si─O─Si bonds, was proposed with each Si atom bonded to a hydrogen atom.^[^ ^12^ ^]^ Subsequently, X‐ray structural analyses were conducted on a siloxene obtained from the reaction of CaSi_2_ with aqueous HCl at ambient temperature, followed by drying over P_4_O_10_.^[^ ^13^ ^]^ A siloxane structure was reported in which the honeycomb silicon framework terminates with hydrogen (─H) and hydroxyl (─OH) substituents, evidenced by alterations in lattice constants. More recently, hydrogen‐terminated silicene, namely layered polysilane (Si_6_H_6_)_n_ was derived from the reaction between CaSi_2_ and concentrated HCl solution at ‐30 °C.^[^ ^14^ ^]^ Alternative methods for the synthesis of silicene nanosheets employing liquid oxidation and exfoliation of CaSi_2_ were also reported.^[^ ^15^, ^16^ ^]^ This technique utilizes iodine as the oxidant, due to its mild oxidizing properties and acetonitrile as a solvent, being compatible with both reactants and by‐products. While enabling large‐scale synthesis, this method requires a very long reaction period (up to 3 weeks).

All these methodologies involve rigorous reaction conditions, characterized by notably low temperatures (e.g., −30 °C) and prolonged reaction durations (5 days), thereby limiting the production efficiency and soliciting the demand for cost‐effective and time‐efficient synthetic protocols, essential for sustainability and fostering technological progress.

In our approach, by strategically alternating vacuum and nitrogen cycles (VANS), we efficiently remove the produced hydrogen, boosting the overall reaction kinetics, while avoiding oxidation under a nitrogen atmosphere. Additionally, unlike conventional methods that often necessitate extreme temperatures, VANS operates effectively at room temperature, offering a more efficient and practical solution for the derivation of microscale silicene flakes.

As a result, employing the VANS method, a significant reduction in synthesis time, transitioning from days to just a few minutes is achieved, providing a viable chemical route for the cheap and quick synthesis of silicene with respect to the expensive physical vapor deposition methods.

## 2 Results and Discussion

The chemical process of silicene formation through topotactical deintercalation follows the reaction:

(1) $$\mathrm{nCaS}i_{2}+2\mathrm{nHCl}\to 2{\left(\mathrm{Si}\right)}_{n}+\mathrm{nCaC}l_{2}+nH_{2}$$

**Figure 1:**
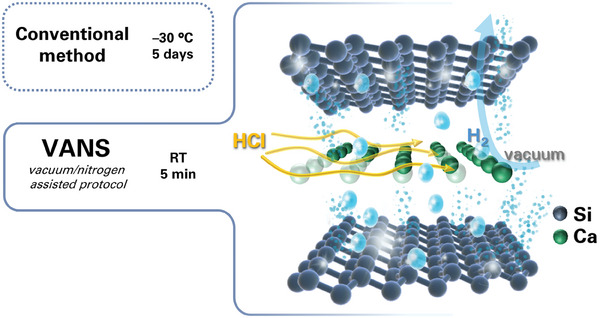
Schematic representation of CaSi_2_ topotactical deintercalation through conventional and VANS method.

However, it's essential to note that the resulting silicene can exhibit various forms or structures, such as silicane (S_6_H_6_) or siloxene (Si_6_H_3_(OH)_3_), depending on the reaction conditions. Regardless of these conditions, the reaction between CaSi₂ and HCl invariably yields hydrogen gas. Our proposed method takes advantage of this hydrogen evolution by employing a vacuum system to continuously remove the produced hydrogen, thereby boosting the reaction kinetics. Silicene flakes are synthesized by immersing CaSi₂ in concentrated HCl, which facilitates the removal of calcium ions. The VANS process, as illustrated in **Figure** 1, employs alternating vacuum and nitrogen cycles, which lead to a noticeable color change from black to yellow‐green. This transition indicates the complete cessation of the hydrogen evolution, resulting in the formation of VANS flakes, as illustrated in Figure S1 (Supporting Information). By integrating room temperature with alternating vacuum and nitrogen cycles, the reaction process is significantly improved while ensuring safety. The vacuum system effectively removes hydrogen gas as it is generated, which accelerates the reaction, reduces pressure buildup, and aids in the desorption of calcium ions from the interlayers. Meanwhile, the nitrogen atmosphere provides an inert environment that enhances the diffusion of HCl into the interlayer regions and prevents oxidation.

For a comparative study, we produced also silicene flakes made by topotactical deintercalation under a conventional method (5 days, −s30 °C).^[^ ^17^ ^]^

The conditions employed in the conventional method were already demonstrated to be optimal for achieving hydrogen‐terminated silicene, commonly known as silicane or layered polysilane.^[^ ^18^ ^]^ Previous studies have also established that deintercalation temperatures above 0 °C result in notable oxygen incorporation, yielding siloxenes (Si_6_H_3_(OH)_3_),^[^ ^19^ ^]^ while temperatures below −20 °C yield nearly 80% hydrogen termination.^[^ ^17^ ^]^ Our approach shows that despite temperatures reaching up to 80 °C due to the exothermic reaction, the impact on the structure of silicene is less significant than previously thought. This is evidenced by the vacuum‐assisted method of producing hydrogen‐terminated silicene nanosheets, similar to silicane, with relatively low oxygen content.

**Figure 2:**
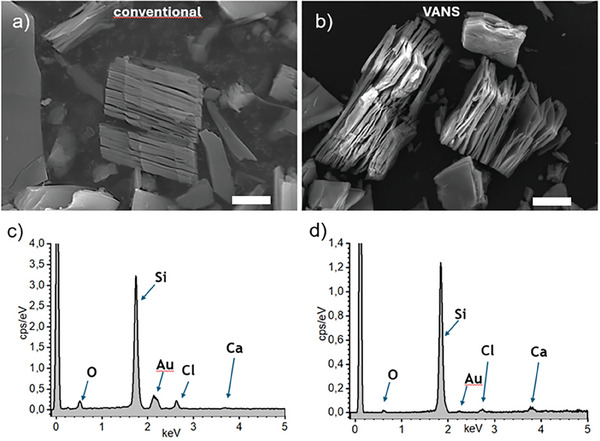
Scanning electron microscopy (SEM) images of representative areas of silicene nanosheets obtained with the classical procedure for 5 days at −30 °C a), and obtained with the VANS method b); corresponding EDX spectra in c), d). Scale bars in SEM images are 20 µm.

Through our optimized protocol, the conversion of CaSi_2_ to silicene flakes demonstrated a yield of ≈94%, similar to the yield obtained through the conventional method, and the resultant materials featured multilayer silicene. The scanning electron microscopy (SEM) images of the silicene nanoflakes obtained via classical or VANS method reveal sample morphologies of multilayer stacked flakes and are reported in **Figure** **2**a,b, respectively. They are indicative of calcium cation deintercalation consistent with previous reports.^[^ ^17^ ^]^ The resultant products, following the topochemical reaction, exhibit a marked divergence from the initial material, CaSi_2_. Originally appearing as a deep grey polycrystalline powder with an unremarkable layered structure (Figure S2, Supporting Information), CaSi_2_ undergoes a structural transformation into a yellow powder with a stacked layered structure while reacted with HCl, yielding well‐exfoliated multilayer silicene nanosheets. The typical lateral size of the multilayered grains ranges from 2–3 µm to ≈100 µm. Their thickness varies with the number of layers forming the grain, with most of them being less than 10 µm thick. However, some multilayered flakes can reach up to 90 µm in thickness.

To confirm the complete removal of calcium from CaSi_2_ in the silicene product, energy‐dispersive X‐ray (EDX) mapping was conducted on both CaSi_2_ and multilayer silicene nanoflakes. The EDX map of CaSi_2_ (Figure S2, Supporting Information) exhibits strong signals from calcium and silicon, indicating a composition that is closely aligned with the stoichiometric one. In contrast, the EDX mapping of multilayer silicene nanoflakes produced via both conventional and topotactical deintercalation (Figure 2c,d, respectively) reveals only trace amounts of residual calcium atoms. The almost identical EDX maps indicate that both processes yield very similar final products.

**Figure 3:**
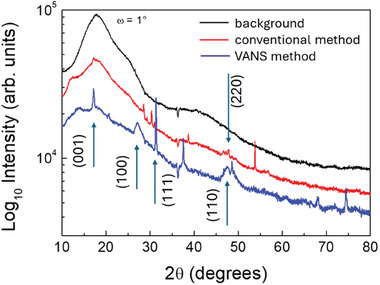
X‐ray diffraction pattern of silicene obtained through conventional (red line) and VANS method (blue line).

The X‐Ray diffraction (XRD) technique is also used to further compare the two different flake powders drop‐cast onto a carbon tape. The corresponding XRD patterns are shown in **Figure** **3**. The variety of collected peaks indicates that the drop‐cast samples are constituted by subpopulations of flakes with different sizes and shapes without a preferred crystallographic orientation. It is worth noting that, for both the de‐intercalated samples, the distinctive peaks of the (001), (100), (111), (110), and (220) crystallographic planes of Si allotropes are detected at 17°, 27°, 31°, 47°, and 48°, respectively. Silicene synthesized by different methods, such as conventional and VANS techniques, can result in variations of crystallographic phases, even with the same chemical composition. These methods lead to distinct atomic arrangements, producing different XRD patterns. For example, the VANS method shows a peak at 38° (210 plane), while the conventional method presents a peak at 51° (311 plane), reflecting differences in crystal growth and orientation.

**Figure 4:**
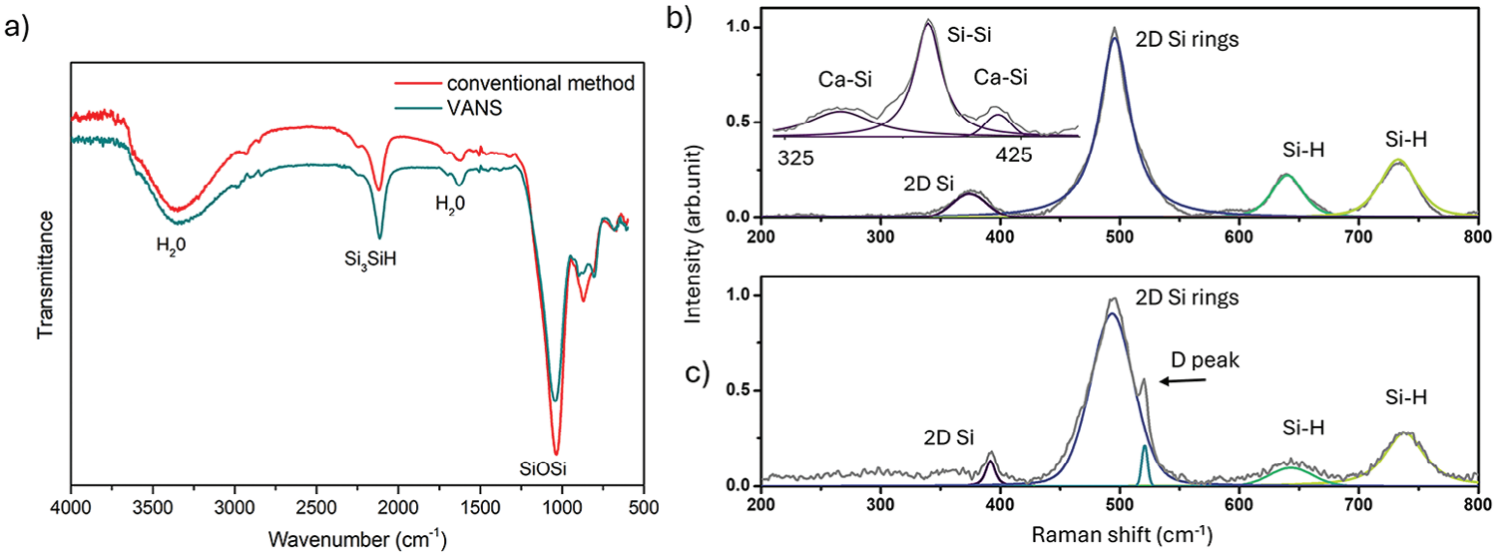
a) FTIR spectra of silicene nanosheets obtained through the conventional (red) and VANS (cyan) method. Raman spectra of silicene nanosheets obtained through the b) VANS and c) conventional method. Inset of panel b): Raman spectra of the CaSi_2_ precursor.

To analyze the molecular structure of the silicene flakes, we employed Fourier transform infrared (FTIR) spectroscopy (**Figure** **4**a) and Raman spectroscopy (Figure 4b,c) techniques.

The FTIR spectra characteristics of silicene, synthesized through either the classical or VANS method, exhibit a remarkable similarity consistent with literature data. In both cases, peaks are observed at 1630 and 3360 cm⁻¹, corresponding to absorbed or intercalated water. Silicon‐hydride modes manifest at 872 and 896 cm⁻¹ (SiH₂), as well as 2100 cm⁻¹ (Si₃SiH). Oxygen‐containing modes are identified at 803 cm⁻¹ (SiO) and 1041 cm⁻¹ (SiOSi), accompanied by weak vibrational modes at 2250 cm⁻¹ (O₃SiH) and 3595 cm⁻¹ (SiOH). Despite the presence of a prominent peak at 1040 cm⁻¹ associated with the SiOSi mode in the FTIR data, it was already demonstrated that the relative intensity of an FTIR feature does not exhibit a direct correlation with the concentration of bonds engaged in that particular mode. Rather, this intensity is directly proportional with the square of the change in dipole moment concerning the normal‐mode coordinate.^[^ ^20^ ^]^ Therefore, the heightened intensity observed in the SiOSi mode (in both samples) can be attributed to the relatively large induced dipole moment of SiO bonds, and not to an increased concentration of SiO bonds. Since this distinctive mode consistently appears in both samples, it is  evident that the oxidation occurs during the work‐up procedure. In both cases, after the completion of the reaction, the obtained flakes are washed several times with dry methanol. In this step of the preparation, a slight oxygenation process is very likely to occur, however, it must be noted that the spectral pattern is very similar to those previously reported in the literature.^[^ ^17^ ^]^

In Figure 4b,c, we examine the congruence of the lattice vibrational characteristics between the CaSi_2_ precursor and the multilayer silicene flakes obtained via the classical method and VANS method by means of Raman spectroscopy. The analysis of the Raman spectra is based on the deconvolution of the signals in terms of Voigt fitting components. Moreover, we stress that the reported spectra are representative of a statistical analysis conducted on a population of more than 30 flakes for both the classical and VANS method.

The Raman peaks observed in the CaSi_2_ precursor exhibit notable frequencies at ≈348 cm⁻¹ (Ca─Si), ≈385 cm⁻¹ (Si─Si), and ≈415 cm⁻¹ (Ca─Si), see inset in Figure 4b. Additionally, Raman spectra acquired from randomly selected flakes in each sample reveal distinct peaks situated at ≈380 and ≈493 cm⁻¹, corresponding to the 2D Si planes and 2D Si6 rings modes, respectively. Additionally, peaks at 636 and 732 cm^−1^ mark the presence of Si and H bonds.^[^ ^21^ ^]^ A direct comparison of the spectra reveals the absence of the Ca‐Si vibrational modes in the spectra of the flakes, demonstrating that the deintercalation of calcium ions is effective in both the adopted approaches. These findings well align with the elemental analysis presented above. An extra peak can be observed at 517 cm^−1^ which is associated with defects or disorder in the crystalline structure of the material. In silicene, extensive theoretical and experimental studies have provided evidence that the D peak is activated due to the presence of defects, that are mainly induced by oxygen atoms interacting with the Si6 rings enabling the in‐plane vibrations of the lattice (breathing modes).^[^ ^22^ ^]^

Notably, the absence of the D peak in the Raman spectra of the silicene flakes obtained through the VANS method represents an indication of high‐quality nanoflakes.

By combining the FTIR and Raman analysis, we conclude that both conventional and VANS methods result mostly in the production of silicene and silicene monohydride terminated (silicane) layers,^[^ ^17^ ^]^ while the small fraction of oxides is to be attributed to the work‐up procedure.

## 3 Conclusion

In conclusion, we have reported on a novel methodology for the topotactical deintercalation of calcium ions from the CaSi_2_ precursor and the formation of silicene nanoflakes. Taking benefit from the introduction of vacuum and nitrogen cycles during the process, the VANS method has enabled the formation of flakes with structural and chemical properties comparable to those produced by the conventional deintercalation methodologies in a sensibly reduced timescale (minutes vs days) and at more controlled experimental condition (room versus low temperature). The presented approach not only allows for a faster production of silicene flakes but also provides a robust and universal approach to the scalable synthesis of other Xenes, such as germanene and stanene from available Zintl phase crystals.^[^ ^21^ ^]^ Its strength lies in its versatility, supporting topotactical deintercalation across a range of Zintl phase materials—such as CaGe₂ and BaSn₂—that share layered honeycomb frameworks composed of p‐block elements. This adaptability makes the VANS method a powerful tool in advancing the synthesis of layered materials. Furthermore, its potential applications extend to diverse fields, including nanotechnology and biotechnology, with implications for photodetectors^[^ ^22^ ^]^ and theranostics,^[^ ^23^ ^]^ underscoring its broad significance across multiple domains.

## 4 Experimental Section

### Materials

CaSi_2_ was purchased from Carlo Erba and HCl (36.5%), NaOH and methanol were purchased from Merck.

### CaSi_2_ Purification

The purification process of the commercial CaSi_2_ involves a pre‐treatment to eliminate potential impurities such as calcium oxides (CaO, Ca_3_SiO_5_) or calcium silicates (Ca_14_Si_19_). The pre‐treatment is performed by immersing CaSi_2_ in a 2 m NaOH solution for 2 h at 60 °C, followed by thorough washing with water until reaching a neutral pH. Subsequently, the purified CaSi_2_ is rinsed with methanol (MeOH) and dried under vacuum.

### Silicene Synthesis via Classical Procedure

Silicene nanosheets were synthesized in the presence of hydrochloric acid. Initially, 200 mg CaSi_2_ was added into a Schlenk and kept in a vacuum for 2 h. Then 20 mL hydrochloric acid aqueous solution (36%) was added. The HCl solution is previously degassed. The siloxene was synthesized under a nitrogen atmosphere, at −30 °C for 5 days. The yellow‐green product was filtered and washed several times with dry methanol. The so obtained silicene was dried under a vacuum and conserved under nitrogen at −20 °C.

### Silicene Synthesis via VANS (Vacuum‐Nitrogen Assisted) Method

Silicene nanosheets were synthesized in the presence of hydrochloric acid. Initially, 200 mg CaSi_2_ was added into a Schlenk and kept in a vacuum for 2 h. Then 20 mL hydrochloric acid aqueous solution (36%) was added. The HCl solutions were previously degassed. The silicene was synthesized by alternating vacuum/nitrogen cycles (10 s vacuum, 10 s nitrogen) for 5 min. Attention: at the beginning, the reaction is very aggressive, due to the high hydrogen evolution, and therefore, when the vacuum cycle is done, very high attention and care are needed. The reaction is finished in a few minutes when the black solid turns to yellow‐green and the hydrogen evolution ceased. For 200 mg of starting material, 8–10 vacuum/nitrogen cycles were required. The yellow‐green product was filtered, and washed several times with dry methanol. The so obtained silicene was dried under vacuum (yield 94%) and conserved under nitrogen at −20 °C.

### Scanning Electron Miscroscopy (SEM)

SEM analysis was performed by using a Phenom Pro Desktop scanning electron microscope (Thermo Fisher Scientific Inc., Eindhoven, the Netherlands), at an accelerating voltage of 15.0 kV, by acquiring the images simultaneously with both back‐scattered and secondary electron detectors, in mixed mode.

### Energy Dispersive X‐ray Spectroscopy (EDX)

Chemical analysis was performed using an X‐Max 50 mm^2^ detector (Oxford Instruments, Abingdon, UK). The samples were sputtered with a thin layer of gold by sputter coater Balzers SCD 040 (Balzers Union Limited, Balzers, Lichtenstein). EDX analysis was performed at a magnification of 300x. For each sample, three areas at different places were measured.

### Powder X‐ray Diffraction (pXRD)

X‐ray diffraction at grazing incidence (GIXRD) angle ω = 1° were performed using an XRD3000 diffractometer (Italstructure) with monochromated X‐ray Cu Kα radiation (wavelength 0.154 nm) and beam size defined by slits aperture of 0.1 × 4 mm.

### Fourier‐Transform Infrared Spectroscopy (FTIR)

Attenuated total reflectance‐Fourier transform infrared (ATR‐FTIR) spectroscopy spectra were recorded on a Nicolet 8700 spectrophotometer (Thermo Fisher Scientific). The spectra were measured in reflectance mode using the ATR (Attenuated Total Reflectance) accessory and were performed in the range of 600–4000 cm^−1^.

### Raman Spectroscopy

The vibrational properties of the flakes were verified by Raman spectroscopy in z‐backscattering geometry using a Renishaw spectrometer (In‐Via) equipped with a solid‐state laser source of excitation wavelength 514 nm/2.41 eV. The laser source is coupled with an optical microscope and objective with numerical aperture = 0.75 and magnification 50×. The laser power on the sample was kept below 1 mW to avoid sample damage.

## Conflict of Interest

The authors declare no conflict of interest.

## Author Contributions

E.K. contributed to conceptualization, methodology, data curation, writing of the original draft, and review and editing. A.E.A. contributed to investigation and data curation. F.G. was involved in investigation, data curation, and writing—review and editing. S.G. contributed to investigation and writing—review and editing. C.G. participated in conceptualization, methodology, and writing—review and editing. A.L. contributed to investigation, data curation, and writing—review and editing. C.M. was involved in investigation and writing—review and editing. C.M. contributed to conceptualization, methodology, investigation, data curation, writing of the original draft, and review and editing. A.M. contributed to conceptualization, investigation, data curation, supervision, funding acquisition, and writing—review and editing.

## Supporting information



Supporting Information

Supplemental Movie 1

## Data Availability

The data that support the findings of this study are available in the supplementary material of this article.
