# Supplementary material for: Vacuum‐Nitrogen Assisted (VANS) Topotactical Deintercalation for Extremely Fast Production of Functionalized Silicene Nanosheets
Source: Small. 2024 Dec 18;21(7):2406088. doi: 10.1002/smll.202406088 (PMC11840468; doi:10.1002/smll.202406088)
Supplement: Supplementary file 1 — Supporting Information [file SMLL-21-2406088-s001.docx]

SUPPORTING INFORMATION

**Vacuum-Nitrogen Assisted (VANS) Topotactical Deintercalation for Extremely Fast Production of Functionalized Silicene Nanosheets**

Erika Kozma*^a^, Christian Martella*^b^, Anita Eckstein Andicsová^c^, Sepideh Gharedaghi^b^, Alessio Lamperti^b^, Chiara Massetti^b^, Andrej Opálek^d^, Carlo Grazianetti^b^, Francesco Galeotti^a^ and Alessandro Molle^b^


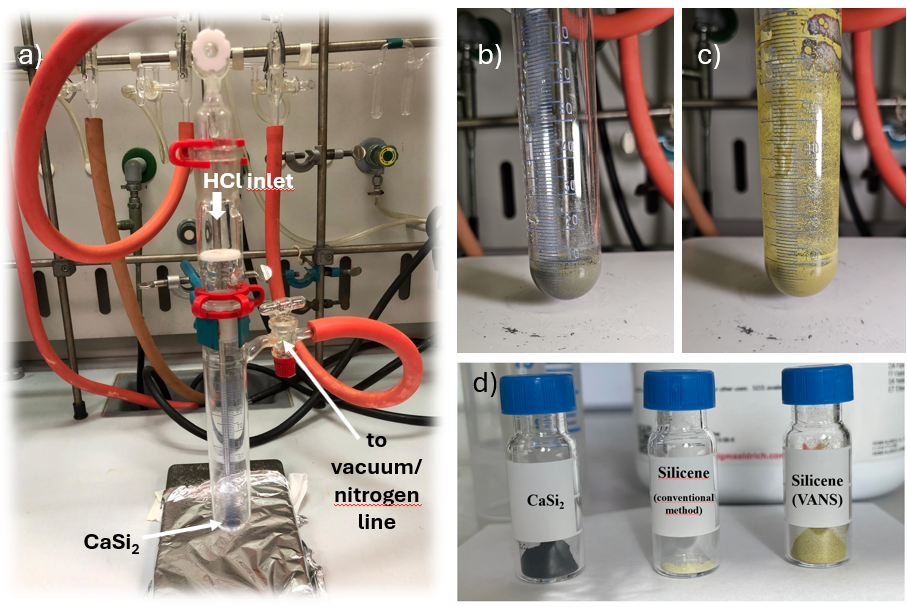


**Figure SI-1.** a) VANS method equipment setup; images of initial (b) and final (c) stage of reaction; d) image of CaSi_2_ (starting material), silicene (conventional method) and silicene (VANS method).

*
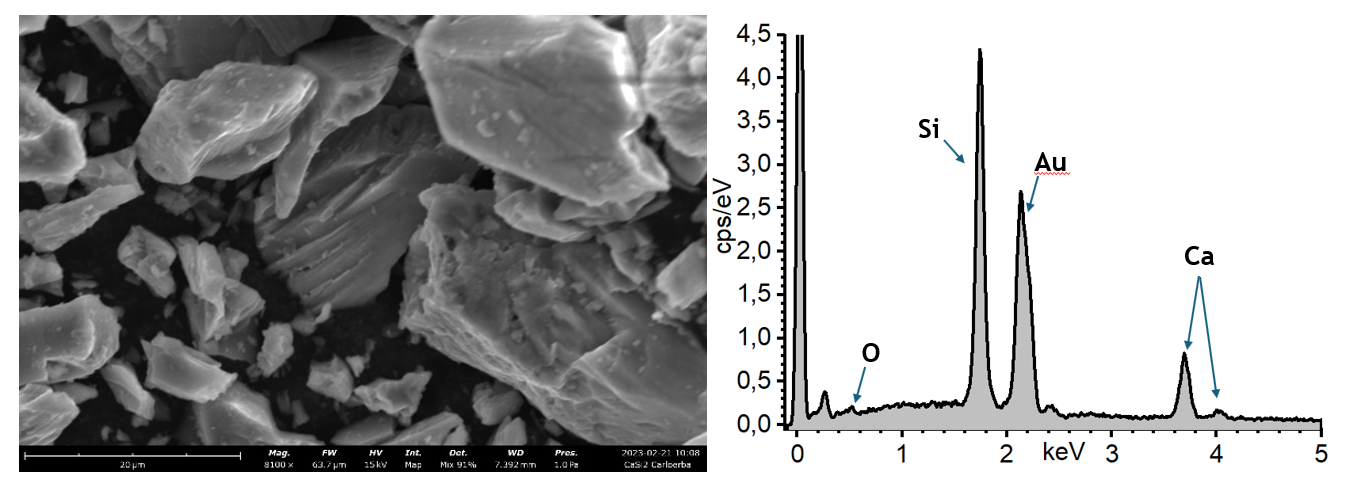
*

**Figure SI-2.** Scanning electron microscopy (SEM) image of representative area of commercial CaSi_2_ (left) and the corresponding EDX spectrum (right). Scale bar in SEM image is 20 µm.
